# HEXA-FC protein therapy increases skeletal muscle glucose uptake and improves glycaemic control in mice with insulin resistance and in a mouse model of type 2 diabetes

**DOI:** 10.1007/s00125-025-06413-7

**Published:** 2025-03-29

**Authors:** Magdalene K. Montgomery, Sihan Lin, Chieh-Hsin Yang, Krishneel Prasad, Zhi Li Cheng, Jacqueline Bayliss, Michael G. Leeming, Nicholas A. Williamson, Kim Loh, Li Dong, Matthew J. Watt

**Affiliations:** 1 Department of Anatomy and Physiology, School of Biomedical Sciences, Faculty of Medicine Dentistry and Health Sciences, The University of Melbourne, Melbourne, VIC Australia; 2 Melbourne Mass Spectrometry and Proteomics Facility, Bio21 Molecular Science & Biotechnology Institute, The University of Melbourne, Melbourne, VIC Australia; 3 St Vincent’s Institute of Medical Research, Fitzroy, VIC Australia; 4 Walter and Eliza Hall Institute of Medical Research, Parkville, VIC Australia

**Keywords:** Ganglioside, Glucose uptake, Hexosaminidase A, IGF1 signalling, Lipid raft

## Abstract

**Aims/hypothesis:**

Type 2 diabetes is a chronic metabolic disorder characterised by insulin resistance and sustained hyperglycaemia, and is a major cause of blindness, kidney failure, heart attacks and stroke. Our team has recently identified hexosaminidase A (HEXA) as an endocrine factor secreted by the liver that regulates sphingolipid metabolism in skeletal muscle. Specifically, HEXA converts GM2 to GM3 gangliosides within cell-surface lipid rafts. Remodelling of ganglioside composition by HEXA enhances IGF1 signalling in skeletal muscle, increasing muscle glucose uptake and improving blood glucose control.

**Methods:**

We produced a long-acting HEXA-FC fusion protein (murine HEXA and the fragment crystallisable [FC] region from IgG1) and evaluated the effects of chronic bi-weekly HEXA-FC administration (1 mg/kg body weight) on glycaemic control in C57BL/6 mice with diet-induced obesity and insulin resistance and the *db*/*db* mouse model of severe type 2 diabetes. Outcome measures included glucose and insulin tolerance, including a stable isotope-labelled GTT and assessment of tissue-specific glucose disposal, as well as proteomics analysis to define changes in skeletal muscle metabolism.

**Results:**

Chronic administration of a long-acting recombinant HEXA-FC fusion protein led to improvements in random blood glucose, fasting blood glucose and glucose tolerance, driven by increased glucose disposal into skeletal muscle, effects that were associated with enhancement of IGF1 signalling in muscle.

**Conclusions/interpretation:**

Given that skeletal muscle is a primary site of insulin resistance in individuals with type 2 diabetes, HEXA-FC protein therapy may open new avenues for therapeutic advancement in type 2 diabetes.

**Graphical Abstract:**

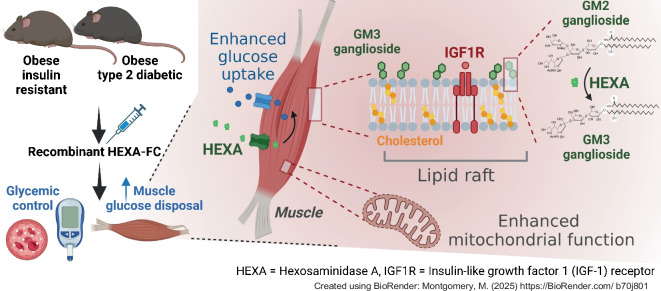

**Supplementary Information:**

The online version contains peer-reviewed but unedited supplementary material available at 10.1007/s00125-025-06413-7.


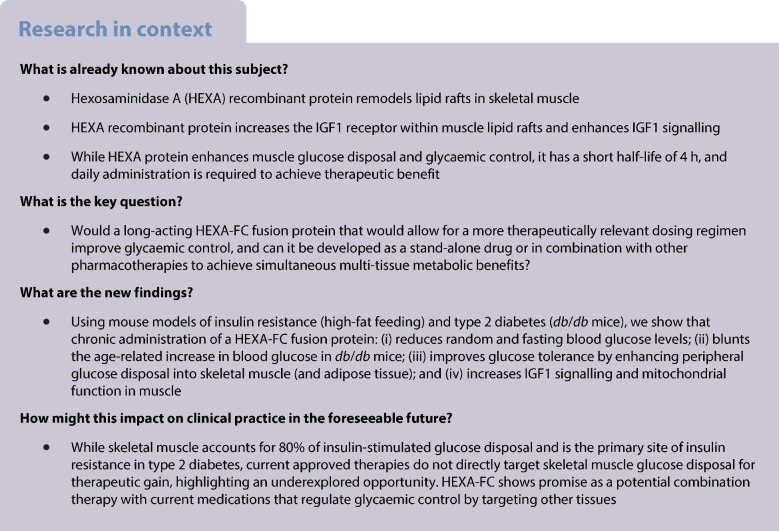


## Introduction

Diabetes has a global prevalence of 10%, and was responsible for 6.7 million deaths in 2021 [1]. Current type 2 diabetes therapies are mainly targeted to adipose tissue (e.g. thiazolidinediones), the intestine (e.g. α-glucosidase inhibitors), the liver (e.g. metformin), the kidney (gliflozins) and/or the pancreas (e.g. sulfonylureas, glucagon-like peptide 1 receptor agonists, dipeptidyl peptidase-4 inhibitors). Importantly, while skeletal muscle accounts for approximately 80% of insulin-stimulated glucose disposal [2], and is the primary site of insulin resistance in type 2 diabetes [3], none of the current pharmacotherapies specifically target skeletal muscle to enhance glucose disposal.

We have recently identified hexosaminidase A (HEXA) as an endocrine regulator of muscle glucose metabolism and as a potential therapeutic target for glycaemic control [4]. HEXA is part of a heterodimer termed β-hexosaminidase A (HexA), which consists of two uniquely functioning subunits, the α subunit (encoded by the *HEXA* gene) and the β subunit (encoded by the *HEXB* gene). While both subunits have the capability to cleave GalNAc residues, only HEXA catalyses the conversion of GM2 to GM3 gangliosides [5], which are sialic acid-containing glycosphingolipids with an oligosaccharide side chain that are present ubiquitously across many tissues, but are particularly enriched in neural tissue [6].

Within the brain, but also other metabolic tissues, including skeletal muscle, gangliosides are primarily localised to lipid rafts [7], which are microdomains on the cell surface that are enriched in cholesterol, glycated sphingolipids and various membrane proteins, thereby playing indispensable roles in many biological processes. These range from regulating nutrient uptake, including the uptake of lipids through CD36 [8] and carbohydrates through GLUT4 [9], and cell signalling [10]. Specifically, lipid rafts have been implicated in regulating signalling through the insulin receptor [11] and the IGF receptor [12], highlighting an essential role for lipid rafts in glucose metabolism and glycaemic control.

While HEXA is primarily localised to lysosomes, an N-terminal signal peptide in HEXA suggests that it is a classically secreted protein. Indeed, we have recently shown that HEXA is secreted from the liver, and that circulating levels are increased in mice with insulin resistance and a mouse model of type 2 diabetes [4]. Increasing plasma HEXA in obese mice with insulin resistance and/or type 2 diabetes improved glycaemic control through HEXA-mediated enhancement of IGF1 signalling and glucose uptake in skeletal muscle [4]. Here, we designed a long-lasting HEXA-FC fusion protein, and assessed the metabolic impact of chronic administration in mice with diet-induced obesity and insulin resistance and in a mouse model of severe type 2 diabetes.

## Methods

### Animal procedures

Mouse experiments were approved by The University of Melbourne Ethics Committee (ID 2021–22307−22642), and conformed to the National Health and Medical Research Council of Australia guidelines regarding the use of animals. Male C57BL/6 mice aged 6–8 weeks were obtained from the Animal Resources Centre (Canning Vale, Australia), while male homozygous *db*/*db* mice (BKS.Cg-*Dock7m*^+/+^ *Lepr*^db^/J; RRID: IMSR_JAX:000700) were bred in-house. Mice were maintained in a temperature-controlled room (22 ± 1°C) with a 12 h light/dark cycle, and had ad libitum access to food and water. C57BL/6 mice were fed a diet enriched in fat and sucrose (43% of energy from fat; SF04-001; Specialty Feeds, Glen Forrest, Australia) for 12 weeks, starting at 8 weeks of age, while *db*/*db* mice were maintained on standard chow.

The HEXA-FC conjugate was produced by Gene Universal (Newark, NJ, USA) using an expression vector containing a secretory leader sequence followed by murine HEXA and the fragment crystallisable (FC) region from IgG1 (hinge region, CH2 and CH3 constant regions) [13]. Mice received HEXA-FC or FC (1 mg/kg body weight) through i.p. injection twice weekly for a total of 8 weeks. Mice were randomised to receive either HEXA-FC or FC, with equal numbers of mice per treatment group within each cage (e.g. two FC-treated and two HEXA-FC treated mice per cage). All experiments were carried out in a non-blinded manner. All outcome measures and experiments performed on these mice are reported in this manuscript.

### Body composition and energy expenditure

Fat and lean mass were measured using time-domain NMR relaxometry (MiniSpec LF50, Bruker). Food intake, locomotor activity, energy expenditure and the respiratory control ratio were assessed using the Promethion system (Sable Systems International) after 12 h of acclimatisation.

### Assessment of glycaemic control

Mice were fasted for 4 h, then received glucose orally (1 or 2 g/kg body weight for *db*/*db* and C57BL/6 mice, respectively) or an i.p. insulin injection (1 or 2 units/kg for C57BL/6 and *db*/*db* mice, respectively). Blood glucose was measured at regular intervals (Accu-Check, Roche), and plasma insulin was assessed at 0, 15 and 30 min (ultra-sensitive mouse insulin ELISA, Crystal Chem, #90080) during the GTT. For assessment of peripheral glucose disposal, mice were fasted for 4 h, then injected i.p. with glucose (as above) and 0.37 MBq 2-[1,2-^3^H(N)]-deoxy-d-glucose (2DG) per mouse (PerkinElmer). Tissue-specific 2DG uptake was determined as described previously [14].

For the stable isotope-labelled GTT, mice were fasted for 4 h, then received an oral load of 6,6-^2^H-glucose (1 or 2 g/kg body weight for *db*/*db* and C57BL/6 mice, respectively; Sigma Aldrich). Blood was collected at intervals after glucose administration, and plasma was used for mass isotopomer analysis of plasma glucose via GC-MS, as described previously [15, 16].

### Assessment of lipid and glucose metabolism

The extensor digitorum longus (EDL) and soleus muscle were excised, and lipid and glucose metabolism assessed, as previously described [4, 17].

### HEXA-FC in plasma and tissues

Plasma HEXA (Abbexa, abx254518) and plasma FC (Mabtech, 3850-1AD-6) were measured by ELISA. Immunoblotting analysis was conducted as described previously [18]. The HEXA antibody (A5646, ABclonal; RRID: AB_2766406), Akt antibody (#9272, Cell Signaling Technology; RRID: AB_329827) and Akt S473 antibody (#9271, Cell Signaling Technology; RRID: AB_329825) were used at a dilution of 1:1000 in TBS containing 0.05% Tween 20 (TBST) and 4% BSA. The HEXA antibody has been previously validated [19]. The secondary antibody (rabbit IgG HRP, NA934V; Sigma Aldrich) was used at a dilution of 1:1000 in TBST containing 4% BSA.

### Gene expression analysis

RNA was extracted using TRI-reagent (Sigma Aldrich), the DNA was degraded using a DNA-free kit (Thermo Fisher) and the RNA was reverse transcribed using iSCRIPT reverse transcriptase (Invitrogen). RT-qPCR was performed using the Taqman system and the Mm01255747_g1, Mm01255770_g1, Mm00440359 and Mm01319006 probes (Thermo Fisher).

### Pancreatic islet isolation

Pancreatic islets were isolated and cultured as described previously [20]. Islets were incubated with vehicle (saline; 154 mmol/l NaCl) or HEXA at 10 ng/ml for 1 or 24 h, followed by stimulation with 2.8 or 20 mmol/l glucose, and assessment of insulin content in cell lysate and media (as detailed in the ‘Assessment of glycaemic control’ section).

### Lipidomics and proteomics analysis

Quadriceps muscle (approximately 10 mg) and pancreatic islets (10 µg protein) were homogenised in 100 µl 1:1 butanol:methanol containing 5 µl SPLASH II LIPIDOMIX Standard (Avanti Polar Lipids). Samples were vortexed for 1 h at room temperature, centrifuged (14,000 *g*, 10 min, 20°C), and the supernatant was transferred into vials with glass inserts. Samples were analysed using a Vanquish UHPLC linked to an Orbitrap Fusion Lumos mass spectrometer (Thermo Fisher) [21].

To enhance ganglioside identification, additional targeted MS was conducted, and identification was performed using LipidSearch version 5.1.11 (Thermo Fisher). The target list was concatenated using ganglioside targets from previous experiments [22]. Relative quantification of gangliosides was achieved by comparing the LC peak areas of identified gangliosides against the internal standard sphingomyelin (SM) (d36:2D9).

Proteomics analysis was carried out using an Orbitrap mass spectrometer (Thermo Fisher) coupled to a nano HPLC (Ultimate 3000 UHPLC, Thermo Fisher) as described previously [21, 22]. Both Qiagen ingenuity pathway analysis (https://digitalinsights.qiagen.com/IPA) and reactome pathway analysis (https://reactome.org/PathwayBrowser/) were used for assessment of the metabolic pathways and biological processes associated with the changes in the skeletal muscle proteome.

### Statistical analysis

Data were assessed for normal distribution using the D’Agostino–Pearson test. Statistical analysis was performed using an unpaired Student’s *t* test or two-way ANOVA, and means were compared using Bonferroni post hoc analysis. All data are shown as means ± SEM. Significance was set as *p* < 0.05.

Statistical analysis of lipidomics and proteomics results was performed using Perseus version 2.0.11 (https://maxquant.net/perseus/). Values were log_2_-transformed, and principal component analysis and volcano plot analysis were performed to identify group-specific clustering. The replicates were grouped accordingly, and all proteins/lipids that had less than 70% of ‘valid value’ in each group were removed, followed by performing a two-sample *t* test (false discovery rate < 5%) to obtain a list of significantly regulated lipids/proteins.

**Fig. 1:**
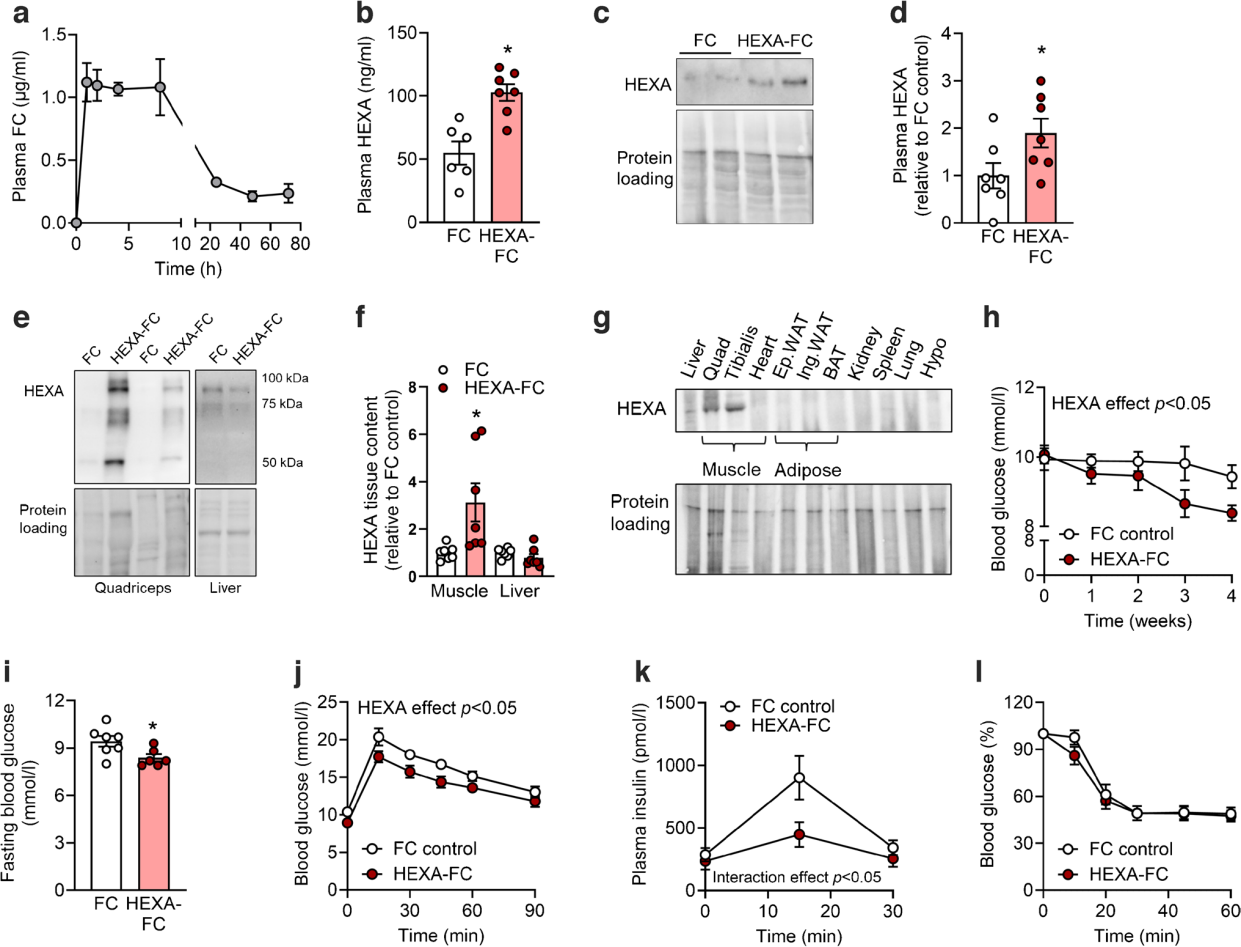
HEXA-FC treatment improves glycaemic control in obese mice with insulin resistance. (**a**) Plasma FC content in lean C57BL/6 mice after a one-off injection with HEXA-FC (1 mg/kg body weight, *n*=5). (**b**–**l**) C57BL/6 mice were fed an HFD for 4 weeks followed by bi-weekly administration of FC or HEXA-FC (1 mg/kg body weight) for a further 8 weeks. Plasma HEXA as assessed by (**b**) ELISA and (**c**, **d**) immunoblotting analysis (*n*=6–7 per group). (**e**, **f**) HEXA protein in quadriceps muscle and liver (*n*=7 per group) and (**g**) tissue distribution of HEXA protein across metabolic tissues. (**h**) Random blood glucose over the last 4 weeks of treatment, (**i**) fasting blood glucose, (**j**) glucose tolerance, (**k**) plasma insulin and (**l**) insulin tolerance of FC control and HEXA-FC treated mice (*n*=6–7 per group). Values are means ± SEM. *p<0.05 vs FC control, as assessed using two-tailed unpaired *t* tests (**b**, **d**, **f**, **i**) or two-way ANOVA and Bonferroni post hoc analysis (**h**, **j**–**l**). Ep.WAT, epididymal adipose tissue; Ing.WAT, inguinal adipose tissue; Hypo, hypothalamus

**Fig. 2:**
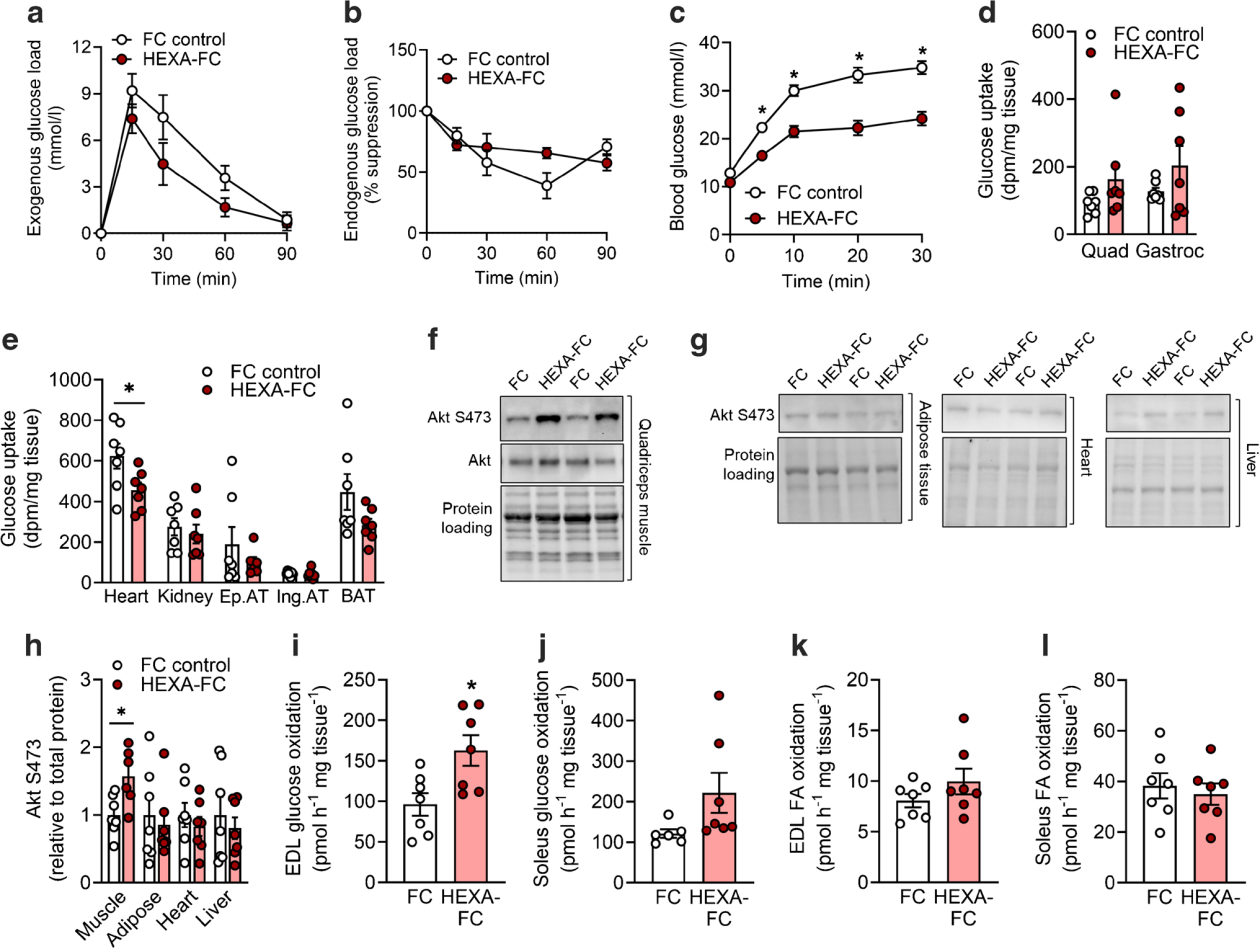
HEXA-FC treatment increases muscle glucose disposal in obese mice with insulin resistance. C57BL/6 mice were fed an HFD for 4 weeks, followed by bi-weekly administration of FC or HEXA-FC (1 mg/kg body weight) for a further 8 weeks. (**a**, **b**) Mice underwent a stable isotope-labelled GTT, followed by assessment of (**a**) exogenous glucose load (HEXA effect, *p*=0.074) and (**b**) endogenous glucose output (*n*=6–7 per group). (**c**–**e**) An i.p. GTT was performed with simultaneous administration of radiolabelled 2DG. Mice were killed 30 min after glucose administration, and tissues were assessed for 2DG uptake: (**c**) glucose tolerance, (**d**) glucose uptake into quadriceps (Quad) and gastrocnemius (Gastroc) muscle (HEXA effect, *p*=0.0616), (**e**) glucose uptake into heart, kidney, epididymal adipose tissue (Ep.AT), inguinal adipose tissue (Ing.AT) and brown adipose tissue (BAT) (*n*=7 per group). (**f**–**h**) Akt S473 phosphorylation in (**f**) quadriceps muscle and (**g**) epididymal adipose tissue, heart and liver; (**h**) quantification of the immunoblots shown in (**f**) and (**g**) (*n*=6–7 per group). (**i**–**l**) Rates of glucose oxidation in (**i**) EDL and (**j**) soleus muscle (*p*=0.092) and fatty acid oxidation in (**k**) EDL and (**l**) soleus muscle (*n*=6–7 per group). Values are means ± SEM. *p<0.05 vs FC control, as assessed using two-tailed unpaired *t* tests (**i**–**l**) or two-way ANOVA and Bonferroni post hoc analysis (**a**–**e**)

No data, samples or animals were excluded from this study, except as follows: Fig. 1b, h, j, 1 × FC control due to lack of sufficient blood/plasma available for analysis; Fig. 2a, 2 × 60 min time point as insufficient blood could be obtained for GC/MS analysis; Fig. 2h, 1 × muscle for HEXA-FC due to insufficient frozen tissue available for analysis; Fig. 2j, k, 1 × FC due to no radioactive counts recorded during analysis; Fig. 4d, weeks 4+5, 4 × *db*/*db* mice not assessed to minimise stress on these highly obese and diabetic mice; Fig. 4h, 1 × HEXA-FC as plasma insulin content could not be detected in ELISA; Fig. 5b, c, all ^3^H tracer analysis for *db/db* mice was carried out in *n*=4 mice per group.

## Results

### Chronic HEXA-FC administration improves glycaemic control in obese mice with insulin resistance

We have recently shown that daily administration of HEXA recombinant protein (1 mg/kg body weight) for 4 weeks in obese mice improves glycaemic control by enhancing glucose disposal into skeletal muscle [4]. While these data are important as proof-of-concept for the therapeutic utility of HEXA, circulating HEXA is rapidly degraded, with plasma levels returning to baseline 4 h following injection [4]. Therefore, we produced a long-acting HEXA-FC fusion protein that is still detected in the circulation 3 days post-injection (Fig. 1a) to allow for more therapeutically relevant dosing.

To evaluate the effects of chronic HEXA-FC on glycaemic control, male C57BL/6 mice were fed a high-fat diet (HFD) for 4 weeks, followed by bi-weekly administration of HEXA-FC or FC (1 mg/kg body weight) for a further 8 weeks. HEXA-FC treatment led to a significant increase in plasma HEXA (Fig. 1b–d), as well as increased HEXA content in quadriceps muscle but not liver (Fig. 1e, f), which is not surprising given that circulating proteins bearing mannose-6-phosphate residues, as is the case for HEXA, can be internalised through the mannose-6-phosphate receptor [23, 24]. Of note, HEXA was not detected in adipose tissue or heart from these mice, which is supported by the finding that HEXA is only expressed in liver and skeletal muscle, but not adipose tissue, heart, kidney, spleen, lung or brain (Fig. 1g).

HEXA-FC treated mice showed a significant reduction in random blood glucose (Fig. 1h), a mild decrease in fasting blood glucose (Fig. 1i) and improved glucose tolerance (Fig. 1j). While fasting plasma insulin was unaffected, there was a significant reduction in glucose-stimulated plasma insulin (Fig. 1k). Insulin tolerance was not impacted by HEXA-FC treatment (Fig. 1l). Together, these data demonstrate that HEXA-FC improves glycaemic control independently of insulin.

### Chronic HEXA-FC increases peripheral glucose disposal into skeletal muscle

To understand whether the improvements in glucose tolerance were due to an increase in peripheral glucose disposal (primarily into muscle) or a suppression of endogenous glucose production/output (primarily by the liver), we investigated glucose homeostasis using a stable isotope-labelled GTT. HEXA-FC treatment tended to increase peripheral glucose disposal (*p*=0.074, Fig. 2a) but did not affect endogenous glucose output (Fig. 2b), indicating a potential impact of HEXA-FC on muscle glucose metabolism. To confirm this, mice underwent an i.p. GTT with simultaneous administration of radiolabelled 2DG (Fig. 2c and electronic supplementary material [ESM] Fig. 1a), which allowed assessment of glucose uptake into tissues. HEXA-FC treatment tended to increase glucose uptake in quadriceps and gastrocnemius muscle (*p*=0.0616, Fig. 2d), but not in adipose tissue or kidney, while glucose uptake was reduced in the heart (Fig. 2e). The reduction in cardiac glucose uptake was independent of changes in markers of cardiac stress/injury (atrial natriuretic peptide [ANP] and b-type natriuretic peptide [BNP]) and pathological cardiac hypertrophy (shift in α- to β- myosin heavy chain) (ESM Fig. 1b). We further observed enhanced Akt S473 phosphorylation in muscle but not adipose tissue, heart or liver (Fig. 2f–h), as well as increased glucose oxidation in EDL muscle (Fig. 2i) and a non-significant increase in soleus muscle (*p*=0.092, Fig. 2j). Fatty acid oxidation (Fig. 2k, l) and fatty acid uptake (ESM Fig. 1c, d) in EDL and soleus muscles did not differ between groups.

HEXA-FC treatment did not impact body weight, fat or lean mass (ESM Fig. 1e–g), any tissue weights (ESM Fig. 1h–k) or whole-body oxygen consumption (ESM Fig. 1l), while the respiratory control ratio was reduced (ESM Fig. 1m), suggesting an increase in systemic fat oxidation, potentially due to an increase in hepatic fatty acid oxidation, as recently shown [22]. Food intake was not different between groups but locomotor activity was mildly reduced (ESM Fig. 1n, o). Together, these results show that HEXA-FC improves glycaemic control by enhancing peripheral glucose disposal into skeletal muscle.

### HEXA-FC treatment increases muscle GM3 and expression of IGF1 target genes

**Fig. 3:**
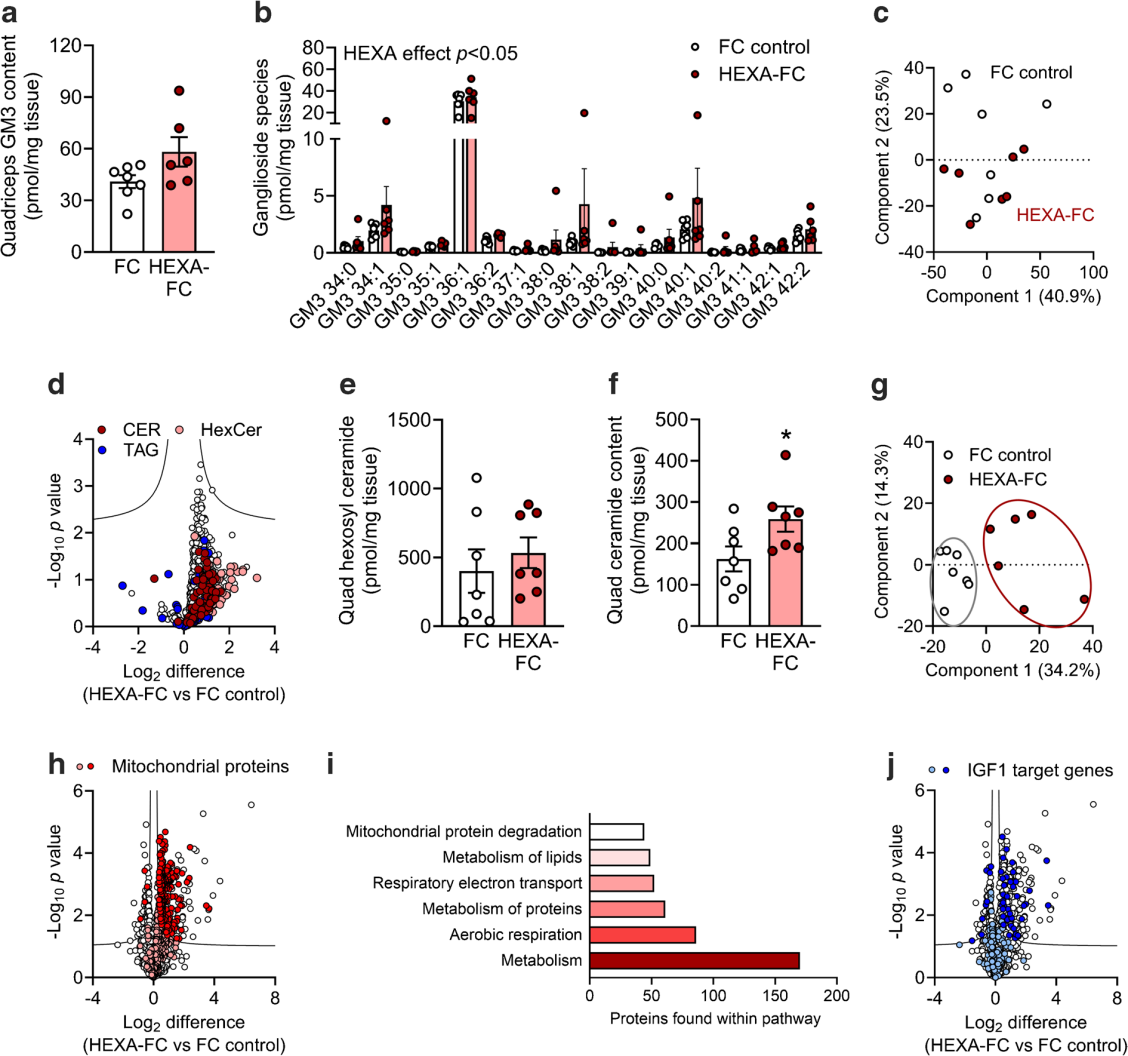
HEXA-FC treatment remodels the skeletal muscle lipidome and proteome. C57BL/6 mice were fed an HFD for 4 weeks followed by bi-weekly administration of FC or HEXA-FC (1 mg/kg body weight) for a further 8 weeks. (**a**, **b**) Total GM3 content (*p*=0.067) (**a**) and GM3 lipid species (**b**) in quadriceps muscle (*n*=6–7 per group). (**c**, **d**) Untargeted lipidomics analysis in quadriceps muscle: (**c**) principal component analysis and (**d**) volcano plot analysis, highlighting non-significant changes in ceramide (CER), hexosyl ceramides (HexCer) and triglycerides (TAG) (*n*=7 per group). (**e**, **f**) Total quadriceps (Quad) muscle content of (**e**) hexosyl ceramides and (**f**) ceramides (*n*=7 per group). (**g**–**j**) Untargeted proteomics analysis in skeletal muscle: (**g**) principal component analysis, (**h**) volcano plot analysis, highlighting mitochondrial proteins, (**i**) analysis of the major pathways associated with proteins increased in muscle following HEXA-FC treatment, and (**j**) volcano plot analysis, highlighting IGF1 targets (*n*=6–7 per group). Values are means ± SEM. *p<0.05 vs FC control, as assessed using two-tailed unpaired *t* tests (**a**) or two-way ANOVA and Bonferroni post hoc analysis (**b**). For the volcano plots in (**h**) and (**j**), dark dots indicate significant changes with HEXA-FC treatment and light dots indicate non-significant changes

We next aimed to elucidate the mechanism underlying the metabolic impact of HEXA-FC on skeletal muscle. Targeted LC/MS ganglioside analysis in quadriceps muscle identified 17 GM3 ganglioside species, with an overall non-significant 42% increase in total GM3 content (*p*=0.067, Fig. 3a) and a significant increase in specific GM3 species following HEXA-FC treatment (Fig. 3b). Untargeted lipidomics analysis identified 1132 lipid species, with no differences between groups (Fig. 3c, d). However, although hexosyl ceramides (direct products of GM3 degradation) were not different (Fig. 3e), we did observe a significant increase in muscle ceramide content (Fig. 3f). This highlights a specific effect of HEXA-FC on sphingolipid remodelling in muscle.

Untargeted proteomics analysis identified 1611 proteins and revealed distinct changes in protein expression in the muscles of mice treated with FC or HEXA-FC (Fig. 3g). Of those, 288 proteins were significantly increased and 64 proteins were reduced following HEXA-FC treatment (Fig. 3h). Interestingly, of the 288 proteins that were increased, 185 proteins were of mitochondrial origin (64%), while only five mitochondrial proteins were reduced in the muscles of HEXA-FC treated mice (Fig. 3h). Supporting these findings, pathway analysis highlighted activation of pathways associated with ‘aerobic respiration’, ‘respiratory electron transport’ and ‘metabolism of proteins and lipids’ (Fig. 3i). We have previously shown that HEXA exacerbates IGF1-mediated signalling, which was associated with increased glucose disposal into muscle [4]. Here, we recapitulated this finding, showing marked upregulation of 47 IGF1 receptor (IGF1R) target genes [25] after HEXA-FC treatment (Fig. 3j). These data highlight that HEXA-FC exacerbates IGF1 receptor-mediated protein expression, which is likely to mediate the observed enhancement of muscle glucose uptake, as previously shown [4].

### Glycaemic improvements with HEXA-FC are recapitulated in a mouse model of type 2 diabetes

**Fig. 4:**
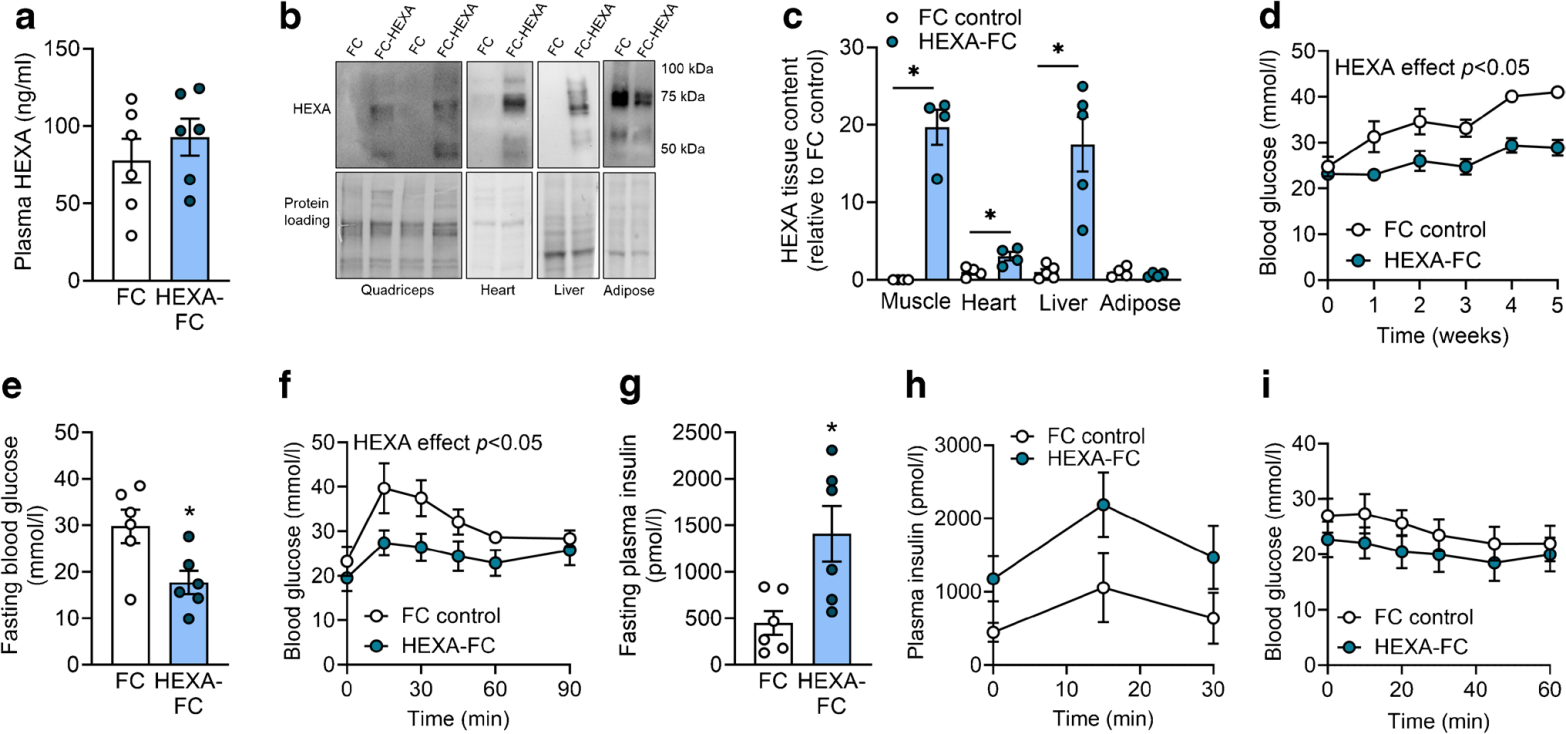
HEXA-FC treatment improves glycaemic control in mice with severe obesity and type 2 diabetes. *db*/*db* mice received bi-weekly administration of FC or HEXA-FC (1 mg/kg body weight) for a total of 8 weeks. (**a**) Plasma HEXA as assessed by ELISA, and (**b**, **c**) immunoblotting analysis of HEXA in quadriceps muscle, heart, liver and epididymal adipose tissue of *db*/*db* mice (*n*=4–5 per group). (**d**) Random blood glucose over the last 5 weeks of treatment, (**e**) fasting blood glucose, (**f**) glucose tolerance, (**g**, **h**) fasting and glucose-stimulated plasma insulin (HEXA effect in **h**, *p*=0.089), and (**i**) insulin tolerance of FC control and HEXA-FC treated mice (*n*=6 per group). Values are means ± SEM. *p<0.05 vs FC control, as assessed using two-tailed unpaired *t* tests (**a**, **c**, **e**, **g**) or two-way ANOVA and Bonferroni post hoc analysis (**d**, **f**, **h**, **i**)

We next assessed the efficacy of HEXA-FC in a mouse model of severe obesity and type 2 diabetes. *db*/*db* mice received HEXA-FC or FC bi-weekly (1 mg/kg body weight) for a total of 8 weeks. While HEXA-FC treatment did not significantly increase plasma HEXA (Fig. 4a), there was a substantial increase in HEXA within skeletal muscle, heart and liver, but not adipose tissue (Fig. 4b, c). Notably, HEXA was barely detectable in any tissue except adipose tissue in mice receiving the FC control.

HEXA-FC treatment led to a pronounced reduction in random blood glucose from a mean of 41 mmol/l in FC-treated mice to 29 mmol/l in HEXA-FC treated mice (Fig. 4d). Importantly, HEXA-FC blunted the age-related increase in blood glucose in *db*/*db* mice (Fig. 4d). HEXA-FC treatment resulted in a 40% decrease in fasting blood glucose (Fig. 4e) and markedly improved glucose tolerance (Fig. 4f). Surprisingly, HEXA-FC increased fasting plasma insulin (Fig. 4g) and tended to increase glucose-stimulated plasma insulin (*p*=0.089, Fig. 4h), but insulin tolerance did not differ between groups (Fig. 4i). Together, these results demonstrate that HEXA-FC treatment improves glycaemic control in a mouse model of severe type 2 diabetes.

### HEXA-FC treatment enhances muscle glucose disposal and increases muscle size in *db*/*db* mice

**Fig. 5:**
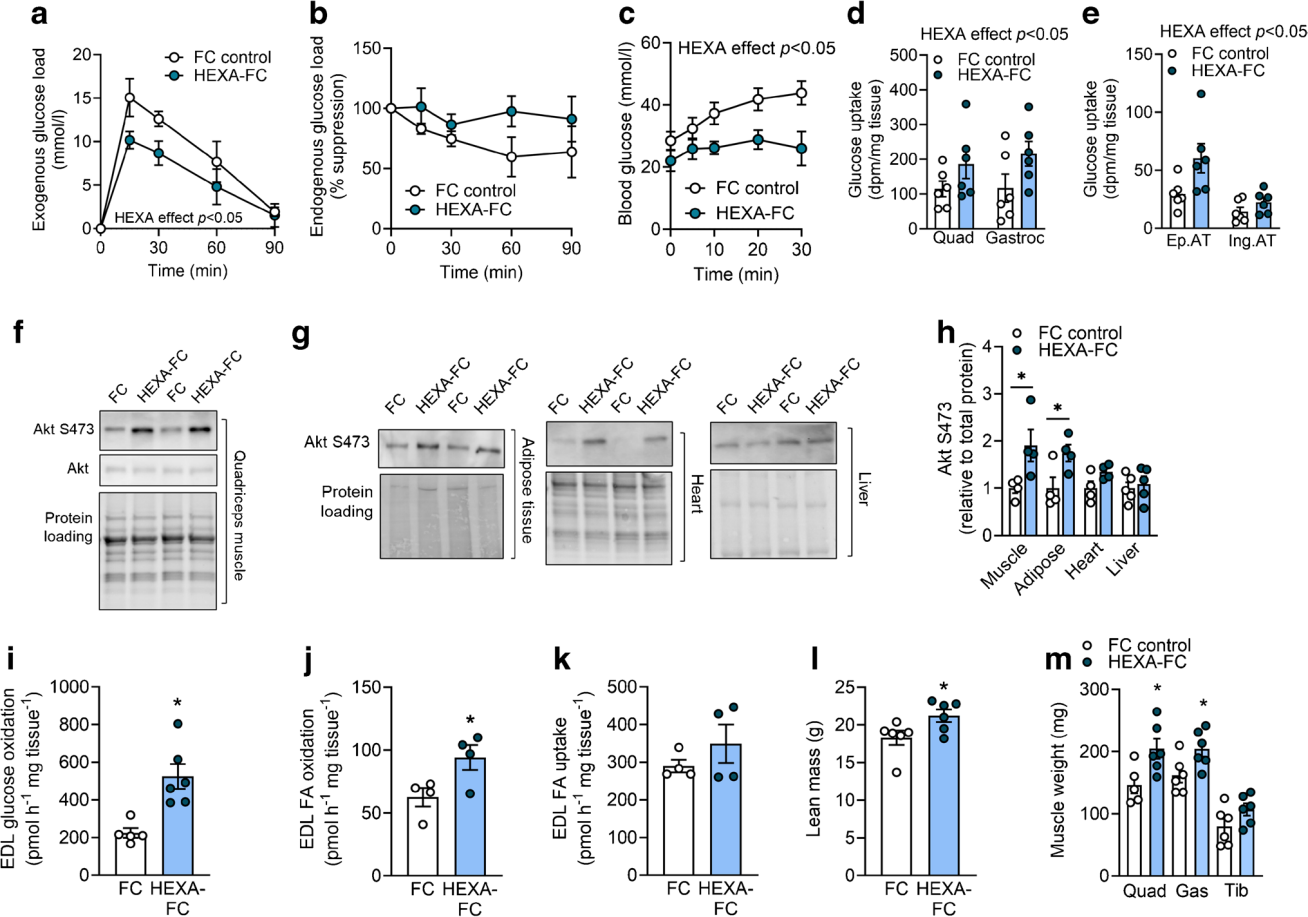
HEXA-FC treatment increases muscle glucose disposal in mice with severe obesity and type 2 diabetes. *db*/*db* mice received bi-weekly administration of FC or HEXA-FC (1 mg/kg body weight) for a total of 8 weeks. (**a**, **b**) Mice underwent a stable isotope-labelled GTT, followed by assessment of (**a**) exogenous glucose load and (**b**) endogenous glucose output (*n*=4 per group). (**c**–**e**) An i.p. GTT was performed with simultaneous administration of radiolabelled 2DG. Mice were killed 30 min after glucose administration and tissues were assessed for 2DG uptake: (**c**) glucose tolerance, (**d**) glucose uptake into quadriceps (Quad) and gastrocnemius (Gastroc) muscle, and (**e**) glucose uptake into epididymal adipose tissue (Ep.AT) and inguinal adipose tissue (Ing.AT) (*n*=5–6 per group). (**f**–**h**) Akt S473 phosphorylation in (**f**) quadriceps muscle and (**g**) epididymal adipose tissue, heart and liver; (**h**) quantification of the immunoblots shown in (**f**) and (**g**) (*n*=4–5 per group; *p*=0.086 for heart). (**i**–**k**) Glucose oxidation (**i**), fatty acid (FA) oxidation (**j**) and fatty acid uptake (**k**) in EDL muscle (*n*=4–6 per group). (**l**) Systemic lean mass and (**m**) individual muscle weights of quadriceps muscle (Quad), gastrocnemius muscle (Gas) and the tibialis anterior (Tib) (*n*=5–6 per group). Values are means ± SEM. *p<0.05 vs FC control, as assessed using two-tailed unpaired *t* tests (**h**–**m**) or two-way ANOVA and Bonferroni post hoc analysis (**a**–**e**)

Using a stable isotope-labelled GTT, we found that HEXA-FC exacerbated peripheral glucose disposal (Fig. 5a) but did not affect endogenous glucose output (Fig. 5b). A GTT with radiolabelled 2DG (ESM Fig. 2a) confirmed a pronounced improvement in glucose tolerance (Fig. 5c), with a concomitant increase in glucose uptake into quadriceps and gastrocnemius muscle (Fig. 5d), as well as epididymal and inguinal adipose tissue (Fig. 5e), but not heart or kidney (ESM Fig. 2b). This was accompanied by increased Akt S473 phosphorylation in muscle, adipose tissue and heart, but not liver (Fig. 5f–h), as well as increased glucose oxidation in EDL muscle (Fig. 5i). Furthermore, HEXA-FC mice exhibited increased fatty acid oxidation (Fig. 5j) but not fatty acid uptake (Fig. 5k) in EDL muscle. While body weight (ESM Fig. 2c) and fat mass (ESM Fig. 2d) were not different, HEXA-FC treatment increased lean mass (Fig. 5l) and the size of individual muscles, including the quadriceps and gastrocnemius muscles (Fig. 5m).

### HEXA-FC treatment increases muscle GM3 content and mitochondrial proteins

**Fig. 6:**
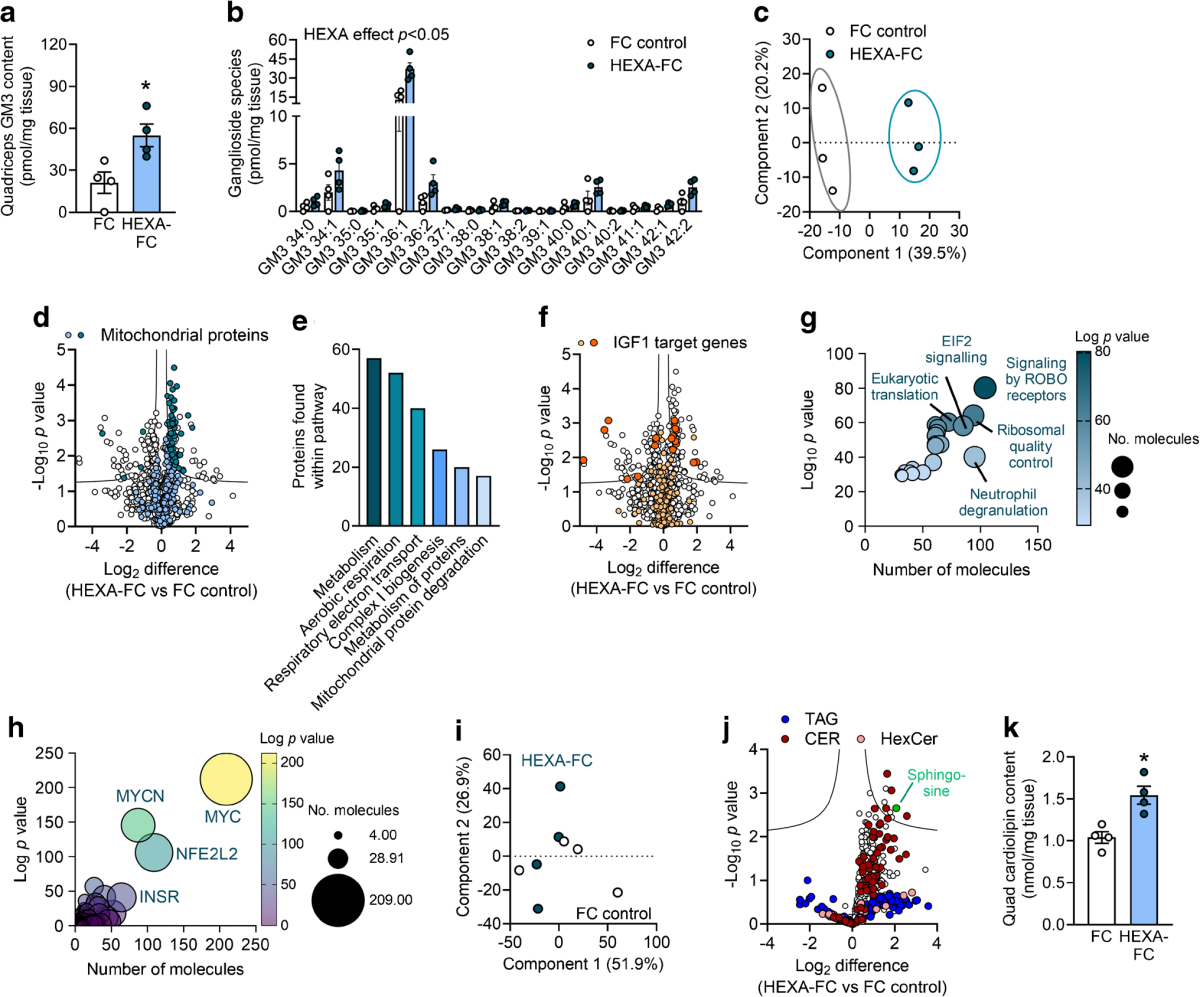
HEXA-FC treatment remodels the skeletal muscle lipidome and proteome in *db*/*db* mice. *db*/*db* mice received bi-weekly administration of FC or HEXA-FC (1 mg/kg body weight) for a total of 8 weeks. (**a**, **b**) Total GM3 content (**a**) and GM3 lipid species (**b**) in quadriceps muscle (*n*=4 per group). (**c**, **d**) Untargeted proteomics analysis in skeletal muscle: (**c**) principal component analysis, (**d**) volcano plot analysis, highlighting mitochondrial proteins. (**e**) Reactome pathway analysis of the major pathways associated with proteins increased in muscle following HEXA-FC treatment. (**f**–**h**) volcano plot analysis highlighting IGF1R targets (**f**), and Qiagen ingenuity pathway analysis of canonical pathways (**g**) and predicted upstream regulators (**h**) (*n*=3 per group). (**i**–**k**) Untargeted lipidomics analysis in quadriceps muscle: (**i**) principal component analysis, (**j**) volcano plot analysis highlighting changes in ceramide (CER), hexosyl ceramides (HexCer) and triglycerides (TAG); (**k**) total cardiolipin content in the quadriceps muscle (Quad) (*n*=4 per group). Values are means ± SEM. *p<0.05 vs FC control, as assessed using two-tailed unpaired *t* tests (**a**, **k**) or two-way ANOVA and Bonferroni post hoc analysis (**b**). For the volcano plots in (**d**) and (**f**), dark dots indicate significant changes with HEXA-FC treatment and light dots indicate non-significant changes. EIF2, eukaryotic initiation factor 2; INSR, insulin receptor; MYC, c-myc; MYCN, n-myc; NFE2L2, nuclear factor erythroid 2-related factor 2; ROBO, roundabout

HEXA-FC treatment significantly increased total GM3 content (Fig. 6a) and GM3 ganglioside species (Fig. 6b). Proteomics analysis identified 1622 proteins, and revealed distinct HEXA-FC mediated changes in the muscle proteome (Fig. 6c, d), particularly a marked increase in mitochondrial proteins (Fig. 6d), with 58 mitochondrial proteins (62%) being significantly increased in HEXA-FC muscle, including subunits of complex I (25 subunits) of the electron transport chain (Fig. 6d). Reactome pathway analysis highlighted activation of pathways associated with ‘aerobic respiration’, ‘respiratory electron transport’ and ‘complex I biogenesis’ (Fig. 6e). Surprisingly, there were only modest changes in IGF1 receptor target genes (Fig. 6f), which may be related to low IGF1 receptor expression in *db*/*db* muscle [26]. However, Qiagen ingenuity pathway analysis revealed that HEXA-FC treatment regulated proteins associated with ‘eukaryotic translation’, ‘eukaryotic initiation factor 2 signalling’ and ‘ribosomal quality control’ (Fig. 6g), pathways that are important for protein synthesis and may contribute to the increase in muscle mass [27]. In addition, upstream regulators predicted to drive these effects included c-myc (MYC), n-myc (MYCN) and the insulin receptor (Fig. 6h), which are known regulators of protein synthesis [28, 29] and could contribute to the increase in muscle mass.

While lipidomics analysis did not point to any substantial changes in the muscle lipidome following HEXA-FC treatment (Fig. 6i, j), we observed a significant increase in the level of cardiolipin, a phospholipid that is localised to the inner mitochondrial membrane (Fig. 6k), suggesting that HEXA-FC treatment may increase mitochondrial capacity in *db*/*db* muscle. In addition, various sphingolipids, including ceramides and sphingosine, were mildly increased in HEXA-FC muscle (Fig. 6j), highlighting mild remodelling of sphingolipid metabolism beyond the expected increases in GM3 gangliosides.

### Acute HEXA treatment inhibits insulin secretion and remodels lipid raft composition within pancreatic islets

**Fig. 7:**
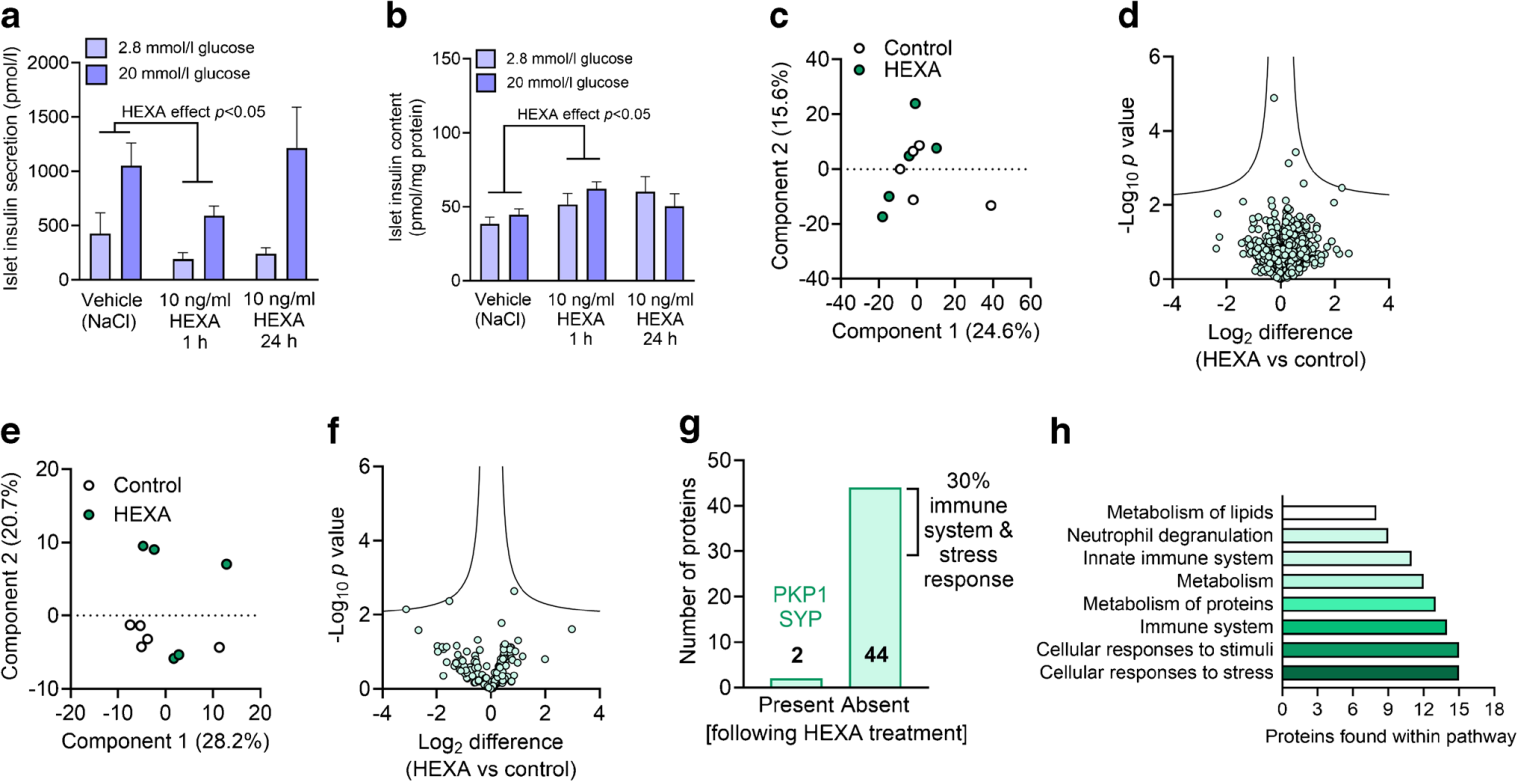
HEXA suppresses insulin secretion and remodels lipid rafts in pancreatic islets. (**a**, **b**) Pancreatic islets were isolated from lean C57BL/6 mice and subjected to acute (1 h) or chronic (24 h) incubation with HEXA protein (10 ng/ml), followed by assessment of glucose-stimulated insulin secretion (**a**) and intracellular insulin content (**b**) (*n*=5 per group). (**c**–**h**) Pancreatic islets were isolated from HFD mice chronically treated with HEXA for 4 weeks, followed by assessment of the proteome of whole islets and fractionated lipid rafts: (**c**) principal component analysis and (**d**) volcano plot analysis of whole islets, and (**e**) principal component analysis and (**f**) volcano plot analysis of fractionated islet lipid rafts; (**g**) proteins that were uniquely present only in control lipid rafts or in lipid rafts of HEXA-treated mice (PKP1, plakophilin-1; SYP, synaptophysin), and (**h**) pathways associated with the proteins that were absent in pancreatic islet lipid rafts following HEXA treatment (*n*=4–5 per group). Values are means ± SEM. A statistically significant HEXA-FC treatment effect (*p*<0.05) is indicated in (**a**, **b**) as assessed by two-way ANOVA and Bonferroni post hoc analysis

HEXA-FC reduced plasma insulin in HFD mice (Fig. 1k) but tended to increase plasma insulin in *db*/*db* mice (*p*=0.089, Fig. 4h). To understand whether HEXA has direct effects on insulin secretion, we isolated pancreatic islets from lean C57BL/6 mice and incubated them with HEXA protein. Interestingly, while HEXA inhibited glucose-stimulated insulin secretion after acute treatment (10 ng/ml, 1 h), this effect was lost after chronic 24 h incubation (Fig. 7a). The acute inhibition of insulin secretion was accompanied by an increase in intracellular insulin content (Fig. 7b).

Given that HEXA inhibited insulin secretion in isolated islets, and following on from our previous observation that chronic HEXA-FC administration reduced plasma insulin in HFD mice, we next took advantage of isolated pancreatic islets obtained from HFD mice chronically treated with HEXA [4], and assessed the proteome of fractionated lipid rafts from these islets using the method described previously [4, 22]. While the whole-islet proteome (3541 proteins identified, Fig. 7c, d) and lipid raft proteome (150 proteins identified, Fig. 7e, f) were similar between groups, we found that 44 proteins were absent from lipid rafts following HEXA treatment (Fig. 7g), and these proteins were associated with the immune system and cellular stress (Fig. 7h), suggesting that HEXA may suppress stress and the immune response within pancreatic islets.

## Discussion

The prevalence of type 2 diabetes is increasing rapidly, with early detection and proactive management being essential for the prevention and alleviation of complications [30]. Here we show that chronic administration of HEXA-FC reduces blood glucose levels and improves glucose tolerance in mice with obesity, mild hyperglycaemia and insulin resistance, and in mice with severe obesity and type 2 diabetes. Mechanistically, we show that HEXA-FC increases skeletal muscle GM3 content, highlighting enzymatic activity within muscle, and enhances glucose disposal into skeletal muscle, while not affecting endogenous glucose production. We further highlight pronounced increases in mitochondrial proteins in muscle across both mouse models, which is associated with an augmentation of muscle glucose and/or fatty acid oxidation, highlighting improvements in skeletal muscle mitochondrial function. Overall, these data support the notion that HEXA-FC has therapeutic potential for treating hyperglycaemia and glucose intolerance.

While lifestyle interventions are commonly prescribed as the first-line treatment in the management of type 2 diabetes [31], many patients require additional pharmacotherapy to achieve glycaemic targets. However, current medications, including metformin, glucagon-like peptide 1 receptor agonists, sulfonylureas and thiazolidinediones (among others) often induce unfavourable side-effects such as hypoglycaemia and weight gain, gastrointestinal distress, increased risk of cardiovascular events, and/or reductions in bone mineral density [30]. Furthermore, despite skeletal muscle accounting for 80% of insulin-stimulated glucose disposal [2], and being the primary site of insulin resistance in individuals with type 2 diabetes [3], current therapies do not directly target muscle glucose disposal for therapeutic gain. Instead, current therapies focus on the pancreas (sulfonylureas, glucagon-like peptide 1 receptor agonists, dipeptidyl peptidase-4 inhibitors), the intestine (α-glucosidase inhibitors), liver (metformin), kidney (gliflozins) or adipose tissue (thiazolidinediones) [32], suggesting that there is an underexplored therapeutic opportunity. Here we show that chronic HEXA-FC administration leads to pronounced improvements in hyperglycaemia and glucose tolerance, which supports our previous findings in obese insulin-resistant mice [4]. Specifically, in *db*/*db* mice that show severe obesity (body weight >50 g) and hyperglycaemia (fasting blood glucose >30 mmol/l), HEXA-FC treatment reduced fasting blood glucose by 12 mmol/l and improved glucose tolerance by 40%, with these effects being greater than those achieved by daily treatment with metformin [13]. Importantly, we show that HEXA-FC targets skeletal muscle glucose disposal, therefore showing promise as a potential combination therapy with current medications that regulate glycaemic control by targeting other tissues.

We have previously shown that HEXA increases IGF1 receptor content within lipid rafts, augments IGF1 signalling, and increases GLUT4 on the plasma membrane, collectively enhancing muscle glucose uptake [4]. The findings were mostly confirmed in the HFD model in this study, with skeletal muscle showing increased HEXA content, enhanced glucose uptake and Akt phosphorylation, as well as increased expression of proteins associated with IGF1-mediated signalling. This is in line with previous studies showing that enrichment of GM3 in the plasma membrane, as would likely be found with increased HEXA, actually impairs insulin receptor-dependent insulin signalling [33], and that mice lacking GM3 synthase have reduced GM3 in skeletal muscle and show enhanced insulin receptor phosphorylation [34]. Combined, these findings suggest that the observed improvements in muscle glucose uptake are not related to increased insulin action in muscle. Instead, we observed increased expression of proteins associated with IGF1 receptor signalling in mice with insulin resistance, and to a lesser extent in *db*/*db* mice, which show dampened IGF1 receptor expression in muscle [26]. While the increase in IGF1 signalling is likely to contribute to the enhanced glucose uptake, conflicting results have been obtained, which indicate that increasing GM3 content suppresses IGF1 receptor signalling [35], but that, in contrast, GM3 depletion promotes IGF1 signalling in keratinocytes [35–37]. Therefore, it is likely that HEXA has a broader role beyond IGF1 signalling. In this respect, we have previously shown that HEXA modulates the localisation of approximately 200 proteins to lipid rafts in myotubes, which was associated with broad remodelling of protein expression [4], and may explain the pronounced increase in proteins associated with improved mitochondrial function and aerobic respiration. This was accompanied by an exacerbation of muscle glucose oxidation, with these metabolic adaptations probably contributing to the enhancements in glucose uptake.

An additional beneficial outcome in this study was a HEXA-induced increase in muscle mass in *db*/*db* mice. HEXA may increase IGF1 signalling in these mice, and IGF1-mediated signalling stimulates muscle growth [38]. Furthermore, proteomics analysis showed HEXA-induced changes in pathways associated with eukaryotic translation and eukaryotic initiation factor 2 signalling, and identified MYC/MYCN as potential transcriptional mediators of HEXA’s action. Given that MYC is a transcriptional regulator of protein synthesis [28], this indicates an alternative mechanism by which HEXA may increase muscle mass in *db*/*db* mice, independently of IGF1. Interestingly, patients with Tay–Sachs disease, who show mutations in the gene encoding HEXA, frequently present with muscle weakness and atrophy [39, 40]. While gene therapy aimed at increasing HEXA levels in individuals with Tay–Sachs disease has undergone early clinical validation [41], to our knowledge, the impact on muscle size/function has not been investigated to date. In addition, several current type 2 diabetes drugs (e.g. semaglutide, dapagliflozin and canagliflozin) that are currently considered for their potential as anti-obesity therapies [42] frequently lead to a loss of muscle/lean mass [43]. This finding further highlights the promise for HEXA as a potential combination therapy with current medications, due to its beneficial impact on both glucose uptake in muscle and on overall muscle mass, with the use of such combination approaches potentially preventing drug-induced loss of lean mass in patients with type 2 diabetes.

Beyond HEXA’s actions on skeletal muscle, we further show that acute, but not chronic, treatment of pancreatic islets with HEXA suppresses insulin secretion, which supports the notion that (i) endocrine HEXA acts on tissues beyond muscle, including the pancreas and liver, as recently shown [22] and (ii) endocrine HEXA has the capacity to directly suppress pancreatic insulin release, as previously reported [4], but that (iii) these changes may be acute and transient, and dependent on the time point of injection, as the *db*/*db* mice in this study did not show the expected suppression of insulin secretion. While we further highlight HEXA-induced lipid raft remodelling within pancreatic islets, future studies are required to fully elucidate the role of HEXA within the pancreas.

Taken together, this study highlights the therapeutic potential of HEXA-FC for the treatment of insulin resistance and type 2 diabetes. Given its pronounced impact on the augmentation of glucose uptake in skeletal muscle, the primary tissue involved in postprandial glucose disposal, the results highlight the potential for HEXA-FC to be developed as a stand-alone drug or in combination with other pharmacotherapies to achieve simultaneous multi-tissue metabolic benefits. Future research is required to optimise the dosage and treatment regimen, and improve protein design to further enhance in vivo stability.

## Supplementary Information

Below is the link to the electronic supplementary material.

ESM Figs (PDF 170 KB)

Supplementary file2 (XLSX 12604 KB)

## Data Availability

An excel file containing all lipidomics and proteomics data has been supplied as supplementary material. All other data, including the MS raw files, are available on request from the corresponding author.

## Abbreviations

2DG: 2-[1,2-^3^H(N)]-deoxy-d-glucose
EDL: Extensor digitorum longus
FC: Fragment crystallisable (region)
HEXA: Hexosaminidase A
HFD: High-fat diet
IGF1R: IGF1 receptor
