# Supplementary material for: HEXA-FC protein therapy increases skeletal muscle glucose uptake and improves glycaemic control in mice with insulin resistance and in a mouse model of type 2 diabetes
Source: Diabetologia. 2025 Mar 29;68(7):1530–43. doi: 10.1007/s00125-025-06413-7 (PMC12176958; doi:10.1007/s00125-025-06413-7)
Supplement: Supplementary file 1 — ESM Figs (PDF 170 KB) [file 125_2025_6413_MOESM1_ESM.pdf]

Montgomery et al. HEXA–FC protein therapy increases skeletal muscle glucose uptake and improves glycaemic control in mice with insulin resistance and in a mouse model of type 2 diabetes

Supplementary Figures

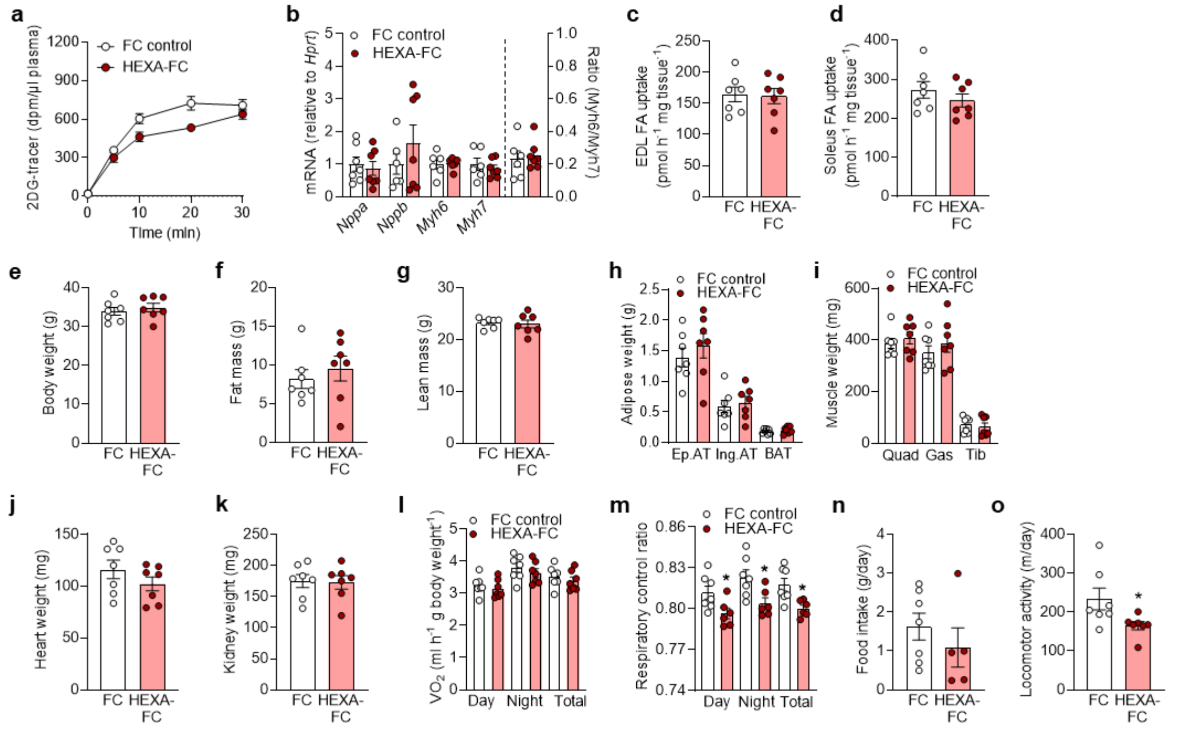

ESM Fig1. HEXA-FC treatment improves glycemic control in obese mice with pre-diabetes. (a) An intraperitoneal glucose tolerance test was performed with simultaneous administration of radiolabelled 2-deoxyglucose (2DG). Mice were killed 30 min after glucose administration and tissues were assessed for 2DG uptake. Shown is plasma tracer appearance (n=7/group). **(b)** Gene expression of cardiac stress and injury markers (n=6-7/group). Fatty acid uptake into **(c)** EDL muscle and **(d)** soleus muscle (n=7/group). **(e)** Body weight, **(f)** fat mass, **(g)** lean mass, **(h)** adipose tissue weights, including epididymal adipose tissue (Ep.AT), inguinal adipose tissue (Ing.AT) and brown adipose tissue (BAT), **(i)** muscle weights, including quadriceps muscle (quad), gastrocnemius muscle (Gas) and tibialis anterior (Tib), **(j)** heart weight, **(k)** kidney weight, **(l)** systemic oxygen consumption, **(m)** the respiratory exchange ratio, **(n)** food intake and **(o)** locomotor activity (n=6-7/group). For all panels, data are means ± SEM. *P < 0.05 vs. FC control, as assessed by two-tailed unpaired t tests (B-K) or 2-way ANOVA and Bonferroni post-hoc analysis (A, L, M). FA, fatty acid; Myh6, cardiac alpha (α)-myosin heavy chain; Myh7, cardiac beta (β)-myosin heavy chain; Nppa, Natriuretic peptide a; Nppb, Natriuretic peptide b

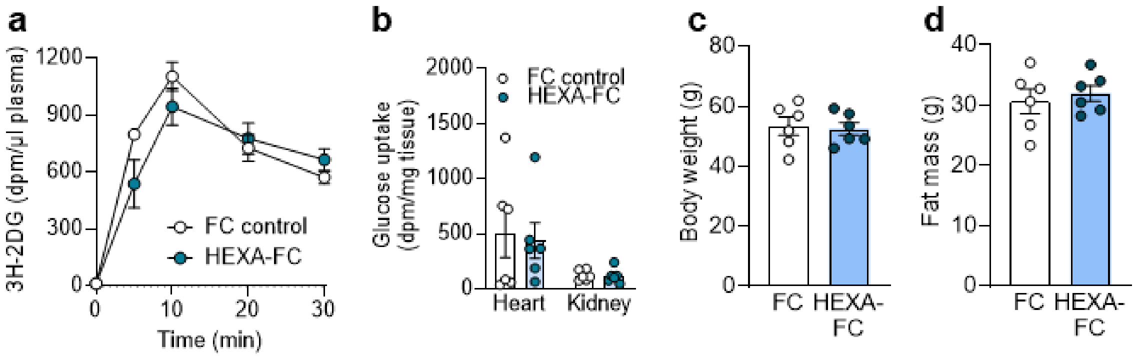

ESM Fig2. HEXA-FC treatment improves glycaemic control in db/db mice with type 2 diabetes. (A) An intraperitoneal glucose tolerance test was performed with simultaneous administration of radiolabelled 2-deoxyglucose (2DG). Mice were killed 30 min after glucose administration and tissues were assessed for 2DG uptake. Shown is **(a)** plasma tracer appearance and **(b)** glucose uptake into heart and kidney (n=6/group). **(c)** Body weight and **(d)** fat mass in db/db mice (n=6/group). For all panels, data are means ± SEM. *P < 0.05 vs. FC control, as assessed by two-tailed unpaired t tests (C-E) or 2-way ANOVA and Bonferroni post-hoc analysis (A).
